# Effectiveness of two different dose administration regimens of an IL-15 superagonist complex (ALT-803) in an orthotopic bladder cancer mouse model

**DOI:** 10.1186/s12967-019-1778-6

**Published:** 2019-01-17

**Authors:** Hideki Furuya, Owen T. M. Chan, Ian Pagano, Chengjun Zhu, Nari Kim, Rafael Peres, Kanani Hokutan, Sarah Alter, Peter Rhode, Charles J. Rosser

**Affiliations:** 1 Translational and Clinical Research Program, University of Hawaii Cancer Center, 701 Ilalo Street Suite 353, Honolulu, HI 96813 USA; 2 Department of Molecular Biosciences and Bioengineering, University of Hawaii at Manoa, Honolulu, HI USA; 3 Pathology Shared Resource, University of Hawaii Cancer Center, Honolulu, HI USA; 4 Cancer Prevention in the Pacific Program, University of Hawaii Cancer Center, Honolulu, HI USA; 5 Altor Bioscience Corp, Miramir, FL USA

**Keywords:** Bladder cancer, Treatment, BCG, IL-15, ALT-803

## Abstract

**Background:**

We set out to determine if the administration of subcutaneous (SQ) ALT-803 was non-inferior to standard intravesical BCG treatment in a carcinogen induced mouse (C57BL/6J) bladder cancer model.

**Methods:**

Using this well-established carcinogen induced mouse model, we studied the effects of various dosing schemas of ALT-803 (SQ alone, SQ with intravesical BCG, intravesical alone, intravesical with intravesical BCG) compared to intravesical BCG alone (positive control) and PBS (negative control). The non-inferiority margin for the difference in bladder weight, as a surrogate for tumor mass, was defined as 7%.

**Results:**

All treatment groups (i.e., ALT-803 SQ alone, ALT-803 SQ with intravesical BCG, ALT-803 intravesical alone, ALT-803 intravesical with intravesical BCG and intravesical BCG alone) demonstrated a significant reduction in tumor burden as evident by bladder weights and H&E stain (p < 0.005). Non-inferiority tests between the intravesical BCG alone group and the additional treatment groups showed that SQ ALT-803 alone (p = 0.04) and BCG plus SQ ALT-803 (p = 0.009) were non-inferior to intravesical BCG alone. In this model, we did not see an appreciable infiltration of CD4^+^ T, CD8^+^ T or CD161/KLRB1^+^ natural killer (NK) cells in the bladder/tumor. When assessing peripheral blood mononuclear cells, SQ ALT-803 alone resulted in a robust induction of CD8^+^ T cells (p < 0.01), NKG2D^+^ NK cells (p < 0.005) and CD3^+^/NKG2D^+^ NKT cells (p < 0.005) compared to other groups, while in splenic tissue, SQ ALT-803 alone resulted in a robust induction of CD3^+^/NKG2D^+^ NKT cells (p < 0.005) compared to other groups.

**Conclusion:**

Subcutaneous ALT-803 treatment alone or in combination with intravesical BCG was well tolerated and was not inferior to intravesical BCG alone. CD8^+^ T, NKG2D^+^ NK and CD3^+^/NKG2D^+^ NKT cell induction along with induction of key cytokines remain steadfast mechanisms behind ALT-803. The enhanced therapeutic index seen with BCG and ALT-803, administered SQ or intravesically, provides a powerful justification for the further development of these regimens.

**Electronic supplementary material:**

The online version of this article (10.1186/s12967-019-1778-6) contains supplementary material, which is available to authorized users.

## Background

In the US alone, the number of new bladder cancer (BCa) cases will be approximately 81,190 (with a preponderance of these patients being men over the age of 50 years) and BCa will result in approximately 17,240 deaths in 2018 [1]. Seventy percent of patients with newly diagnosed BCa present with non-muscle invasive bladder cancer (NMIBC), which is disease confined to the mucosa and submucosal tissues. In patients with NMIBC, the administration of adjuvant intravesical chemotherapy or Bacillus Calmette-Guérin (BCG) often can prolong the progression-free interval after initial tumor resection [2]. Unfortunately, despite these therapies, patients with NMIBC remain at high risk of tumor recurrence (~ 70%), making BCa one of the most prevalent cancers in the US, second only to colorectal cancer [2, 3]. In addition to this high rate of tumor recurrence, 30% of patients with NMIBC will progress to muscle invasive bladder cancer (MIBC), which is associated with a reduced overall survival (5-year survival < 50%) compared to NMIBC (5-year survival > 90%), whilst another 50% of these NMIBC patients will undergo removal of their bladders in an attempt to control their disease [4]. In the management of NMIBC, no new drug has been FDA approved in over 15 years [5].

Recently, we published our results of intravesical administration of ALT-803 (novel IL-15N72D/IL-15Rα-Fc superagonist complex) along with intravesical BCG in a well-established carcinogen induced rat model. In this study we noted: (a) intravesical ALT-803 was safe and well tolerated alone and in combination with BCG, (b) as a single treatment agent, ALT-803 reduced tumor burden by 35% compared to a tumor reduction of 15% with BCG alone, (c) combination of ALT-803 plus BCG reduced tumor burden by 46% compared to control and was associated with natural killer (NK) cell activation [6]. Motivated by these compelling preclinical data related to IL-15, we tested the therapeutic efficacy of intravesical ALT-803 with intravesical BCG in a phase Ib clinical trial in subjects with BCG-naïve NMIBC. The induction treatment regimen was administered according to a previously published Southwest Oncology Group intravesical BCG protocol [7]. Patients had a routine cystoscopy and voided urinary cytology (VUC) every 3 months for 2 years to determine response. We noted that the combination therapy was well-tolerated and all patients were disease-free (i.e., complete response) at 24 months follow-up with no patients experiencing disease recurrence or progression [8].

Promising early phase clinical results with intravenous and subcutaneous ALT-803 administration have been also reported in allogeneic hematopoietic cell transplantation, non-small cell lung cancer, melanoma, renal cell carcinoma and head and neck cancers, suggesting that systemic ALT-803 may have a therapeutic effect on NMIBC [9–11]. To date, no systemic therapy has been proven to be effective in the NMIBC setting, thus there is a need to evaluate systemic ALT-803 therapy given subcutaneously prior to embarking on human clinical trial. In this report, we set out to determine if subcutaneous (SQ) ALT-803 given in the setting of NMIBC was non-inferior to intravesical BCG.

## Methods

### Animals, reagents, and tumor model

Previously we had demonstrated the anti-tumor activity of ALT-803 in a carcinogen induced orthotopic BCa rat model [6]. However, due to limitations of our animal facility as well as the ease of handling mice compared to rats, we used a similar carcinogen induced mouse BCa model in the current project [12]. Ninety female C57BL/6J mice, 4 weeks old, were obtained from Jackson Laboratories (Bar Harbor, ME). Animal care was in compliance with the recommendations of *The Guide for Care and Use of Laboratory Animals* (National Research Council) and approved by our local Institutional Animal Care and Use Committee. Mice were housed and handled in the laboratory animal resources facilities at the University of Hawaii. Mice were maintained under controlled conditions of humidity (50 ± 10%), light (12-h light–dark cycle) and temperature (23 ± 2 °C). *N*-Butyl-*N*-(4-hydroxybutyl) nitrosamine (BBN) was purchased from TCI America (Portland, OR). Lyophilized BCG was purchased from Sanofi Pasteur Limited (Toronto, CA) and reconstituted as per instructions with sterile normal saline. ALT-803 was obtained from Altor Bioscience (Miramar, FL). After allowing animals to acclimate to our facility for 1 week, all animals except nine received 0.05% BBN in their drinking water continuously for 20 weeks which induced the formation of orthotopic BCa [13–15].

### Intravesical treatment

**Fig. 1:**
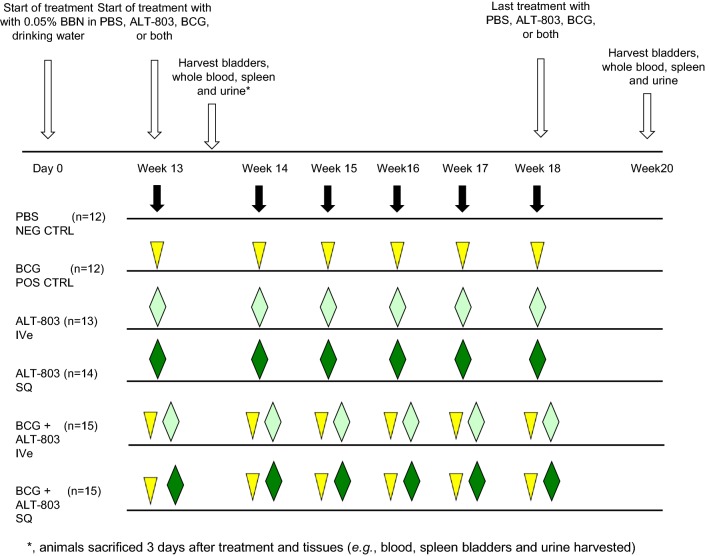
Study treatment schema. Treatment with PBS (negative control), BCG (positive control), intravesical (IVe) ALT-803, SQ ALT-803, IVe BCG + IVe ALT-803 and IVe BCG + SQ ALT-803 commenced on week 13 after 12 week exposure to 0.05% *N*-butyl-*N*-(4-hydroxybutyl) nitrosamine in drinking water. Mice were randomly assigned into six groups and treated weekly for six consecutive weeks according to the schedule. Three mice from each group was sacrificed and necropsied 3 days after the 1st week’s treatment. Then the remaining mice were sacrificed and necropsied on week 20. *CTRL* Control, *IVe* intravesical, *SQ* subcutaneous

At 12 weeks of BBN treatment, mice were randomized to one of six treatment groups (control-PBS; BCG at 135 μg/dose intravesically, ALT-803 at 0.1 μg/dose intravesically, ALT-803 at 0.2 mg/kg SQ, BCG at 135 μg/dose intravesically + ALT-803 at 0.1 μg/dose intravesically or BCG at 135 μg/dose intravesically + ALT-803 at 0.2 mg/kg SQ). Each group contained at least twelve animals. Subcutaneous administration was performed on the neck of the mice with a tuberculin needle and syringe. All bladder instillations were done in 50 μl of sterile PBS. Mice were anesthetized with isoflurane, then a 22-gauge Teflon angiocatheter (transurethral catheter) was placed in the bladder and urine completely drained from the bladder. Intravesical medication was instilled through the transurethral catheter and allowed to dwell in the bladder for 1 h by occlusion of the urethra with a purse string suture. After 1 h, the purse string suture was removed and mice were stimulated to expel bladder contents [16, 17]. Three days after the first treatment, three animals from each group were sacrificed and tissues (whole blood, serum, urine, bladder and spleen) were harvested and processed for subsequent analysis. The ascribed therapy was administered weekly for a total of 6 weeks to the remaining animals. Two weeks after completion of the therapy, animals were sacrificed and tissues (whole blood, serum, urine, bladder and spleen) were harvested and processed for subsequent analysis. Figure 1 illustrates the study treatment schema.

### Histopathology of tumor sections

Resected bladders were initially weighed then filled with 50 µl of 10% neutral buffered formalin. The bladder neck was ligated and the entire specimen was placed in 10% neutral buffered formalin. Bladders in formalin were embedded in paraffin, sectioned (5 µm) and placed on Superfrost plus Micro slides (Fisher Scientific, Pittsburgh, PA). Deparaffinized sections from each mouse were subjected to H&E stain for histological evaluation. Additional deparaffinized slides were treated with 3% hydrogen peroxide in PBS to block endogenous peroxidase activity. Deparaffinized slides were then subjected to citric acid antigen retrieval. Slides were incubated overnight with antibodies against CD4 (eBioscience; 4SM95; rat monoclonal antibody, dilution 1/100), CD8a (eBioscience; 4SM15; rat monoclonal antibody, dilution 1/200), and CD161/KLRB1 (Invitrogen; PA5-50375, rabbit polyclonal antibody, dilution 1/2000). Next, the sections were incubated with biotinylated anti-mouse IgG (H + L) antibodies at 10 µg/ml (Vector Laboratories INC., Burlingame, CA). Subsequently, sections were stained using the Ultra-Sensitive ABC Mouse IgG staining kit (EMD Millipore, Billerica, MA). All stained sections were imaged using a Nikon Eclipse E400 light microscope (Nikon Inc., Melville, NY) with the QIClick™ digital CCD camera (QImaging, Surrey, BC, Canada) and the Nikon NIE-Elements Basic Research imaging software.

Immunohistochemical staining was assessed as previously described [6]. Three representative areas from each specimen were identified at 4× magnification and then images were taken for quantitation of immune-positive cells at 200×. A board-certified pathologist (OC) counted the number of immune-positive cells in each histological field. Cells with questionable nuclear staining were discounted.

### Flow cytometric analysis of PBMC and splenocytes for CD3^+^, CD8^+^, NKG2D^+^ and CD3/NKG2D^+^ cells

Mouse whole blood samples collected 3 days after week 13 and at week 20 were placed in EDTA vacutainers and peripheral blood mononuclear cells (PBMC) were stained by incubation with fluorescently labeled antibodies CD3-FITC (eBioscience) + CD8a-PE (eBioscience) and CD3-FITC + NKG2D-PE (eBioscience) or isotype control antibodies (eBiosciences, San Diego, CA) for 30 min at room temperature, followed by red blood cell lysis using BD FACS Lysing Solution (BD Biosciences, San Jose, CA) at room temperature for 3–5 min. Cells were subsequently washed twice with PBS containing 1% fetal bovine serum. Stained cells were analyzed using a BD LSR Fortessa flow cytometer (BD Biosciences), and data were collected for 10,000 events/sample. Analysis of the data collected was performed using FlowJo software (TreeStar, Ashland, OR). In addition to assessing PBMC, spleens collected 3 days after week 13 and at week 20 were gently homogenized on a petri dish and then passed through a Cellector Tissue Sieve (Bellco Glass, Vineland, NJ) resulting in single cell suspensions. Red blood cells were removed from mouse splenocytes with BD FACS Lysing Solution by incubation for 3–5 min at room temperature, followed by centrifugation at 1200 rpm for 5 min. The pelleted splenocytes were washed twice with PBS containing 1% fetal bovine serum followed by direct immunofluorescence staining as described above.

### Urine and serum cytokine ELISA assay

Voided urine samples from each mouse collected 3 days after week 13 and at week 20 were placed in a tube on ice containing a concentrated urine stabilizer solution (2 M Tris–HCl [pH 7.6], 5% BSA, 0.1% sodium azide, and a COMPLETE™ mini protease inhibitor tablet (Roche Diagnostics, Indianapolis, IN)). Urine was centrifuged and supernatant was stored at − 80 °C. Serum samples collected 3 days after week 13 and at week 20 in vacutainers containing EDTA were centrifuged at 10,000×*g* for 15 min and stored at − 20 °C. Urine and serum samples collected 3 days after week 13 were profiled for 9 cytokines using a customized Luminex assay (R&D Systems, Minneapolis, MN), which measures the expression of CCL5/RANTES, IFNγ, IL1α, IL1β, IL10, IL13, IL5, IL6, and TNFα. Urine samples collected at week 20 were profiled for 20 cytokines using a Cytokine 20-Plex Mouse Panel Luminex assay kit (Thermo Fisher, Waltham, MA), which measures the expression of GM-CSF, IP-10, FGF-basic, IFNγ, KC, VEGF, IL1α, MCP1, IL1β, MIG, IL2, MIP-1α, IL4, IL5, IL6, IL10, IL12 (p40/p70), IL13, IL17, TNFα. Measurements were performed using a Luminex 200 instrument (Luminex Corp., Austin, TX, USA) and were analyzed using a standard curve for each molecule (xPONENT^®^ software, Luminex Corp).

### Statistics

The non-inferiority comparisons for bladder weight were powered for separate tests between intravesical BCG alone and each additional treatment group. We assumed that the frequency with which the primary efficacy outcome would occur would be equal across the five groups. We chose a conservative non-inferiority margin of 7%, which preserves two-thirds of the 95% CI difference between intravesical BCG alone and PBS control.

Experimental data were expressed as mean ± SD, unless otherwise indicated. Statistical analysis was conducted using non-parametric Kraskal–Wallis test followed by post hoc Dunnet’s test for immunostaining of the bladder, urine cytokines and serum cytokines. Parametric ANOVA test followed by post hoc Bonferroni’s test were performed on the data related to lymphocytes within spleen and lymphocytes within the peripheral blood. A *p *< 0.05 was considered significant. All statistical analyses and figures were carried out using GraphPad Prism software 5.0 (GraphPad Software, Inc., La Jolla, CA).

## Results

### Anti-tumor activity of BCG, ALT-803 and combination ALT-803 plus BCG

**Fig. 2:**
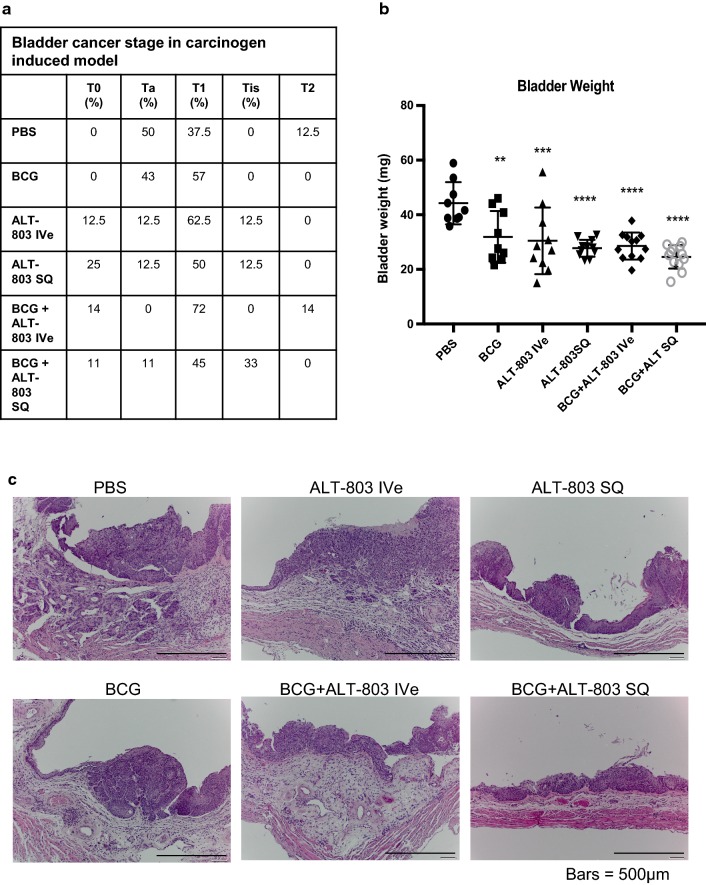
Anti-tumor activity of BCG, ALT-803 and ALT-803 plus BCG. **a** On week 20, mice were sacrificed and resected bladders were fixed in 10% buffered formalin and embedded in paraffin and subsequently stained with H&E. Histopathologic examination of the tumors allowed pathologic stage classification into T0, Ta, T1, Tis and T2. **b** Bladder weight was reduced in all treatment groups compared to PBS negative control. Greatest reduction in bladder weights was seen in the group treated with ALT-803 SQ, BCG + ALT-803 Ive and BCG + ALT-803 SQ (*p* < 0.005). **c** Representative H&E images from each of the six groups are illustrated

Bladder tumors were not present in any animal in the group not exposed to BBN, while 100% of animals treated with BBN in the PBS negative control group (Group 1) were noted to develop tumors. No animals had palpable, i.e., advanced tumors. There was no noticeable treatment related body weight loss nor altered macroscopic and histological observations in any treatment group. The incidence of Ta, T1, Tis and T2 tumors was not different between any treatment groups. Interestingly, the tumor burden (bladder weight) was noted to be significantly different in the treatment groups (Groups 2–6) compared to PBS control. For example, we noted a 28% reduction in mean bladder weight with intravesical BCG alone compared to PBS control (*p* < 0.01), 28% reduction in mean bladder weight with intravesical ALT-803 alone compared to PBS control (*p* < 0.005), 37% reduction in mean bladder weight with SQ ALT-803 alone compared to PBS control (*p* < 0.001), 36% reduction in mean bladder weight with intravesical BCG plus intravesical ALT-803 compared to PBS control (*p* < 0.001) and 44% reduction in mean bladder weight with intravesical BCG plus SQ ALT-803 compared to PBS control (*p* < 0.001). Non-inferiority tests between the intravesical BCG alone group and additional treatment groups showed that SQ ALT-803 alone (p = 0.04) and intravesical BCG plus SQ ALT-803 (p = 0.009) were non-inferior to intravesical BCG alone. All results were subsequently confirmed on thorough histologic examination. Figure 2 depicts the number of T0, Ta, T1, Tis and T2 tumors per group, the mean ± SD of these bladder weights and a representative of corresponding H&E stain.

### Assessment of therapeutic induction of lymphocytes

**Fig. 3:**
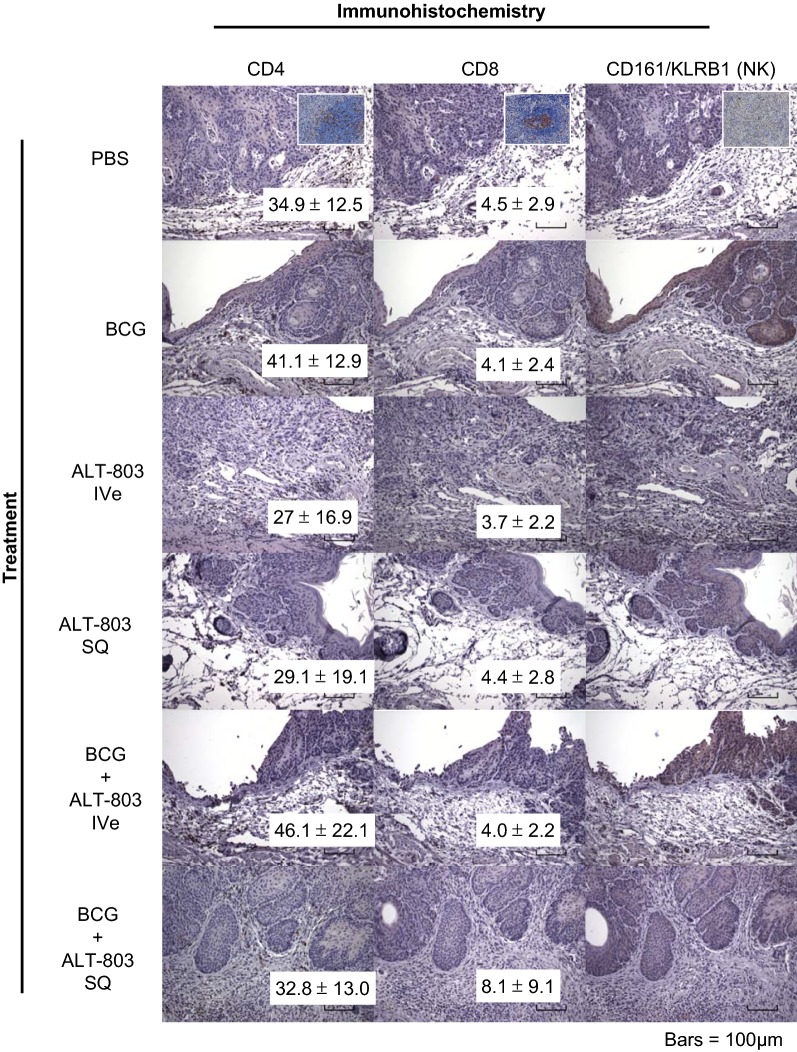
Treatment-related infiltration of lymphocytes in bladders of female mice bearing tumors. No difference in the number of infiltrating CD4^+^ and CD8^+^ T cells was noted in any of the treatment groups compared to PBS control. No CD161/KLRB1 (NK) immune-positive cells were found in any group. Original magnification, ×200. Insert, panel positive and negative control cells from mouse spleen at ×400

**Fig. 4:**
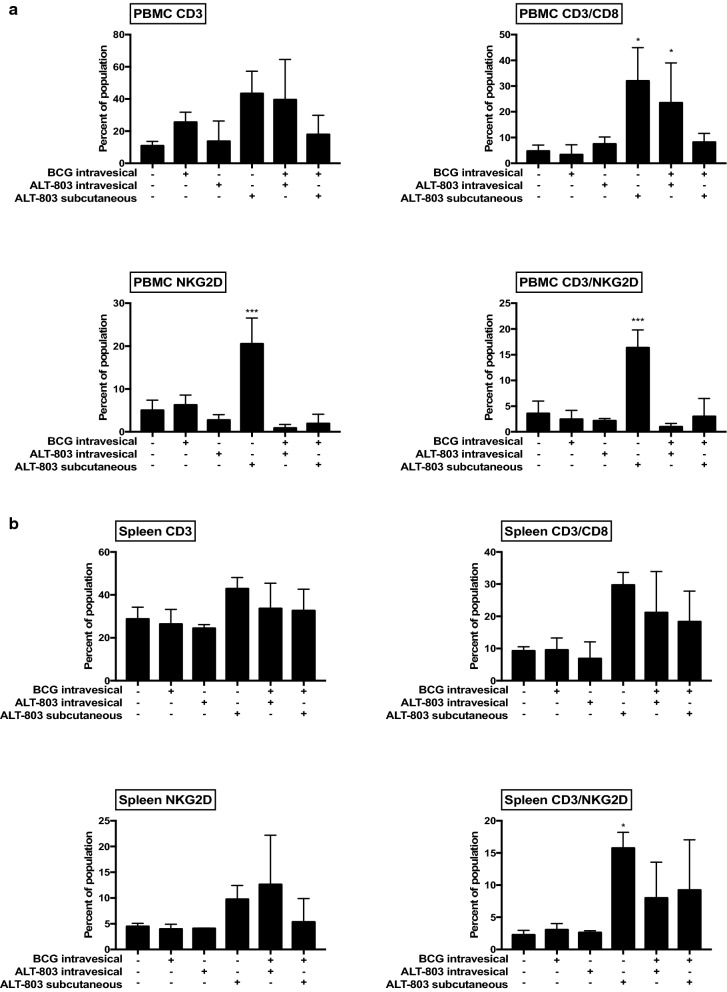
Treatment-related activation of peripheral immune cells in female mice bearing tumors. **a** Peripheral blood mononuclear cells collected 3 days after week 1 treatment were isolated and analyzed by flow cytometry for expression of CD3^+^, CD3^+^/CD8^+^, NKG2D^+^ and CD3^+^/NKG2D^+^ expression. SQ ALT-803 alone resulted in significant increase in CD8, NKG2D and CD3/NKG2D expression cells compared to PBS control. **b** Splenocytes collected 3 days after week 1 treatment were isolated and analyzed by flow cytometry for expression of CD3^+^, CD3^+^/CD8^+^, NKG2D^+^ and CD3^+^/NKG2D^+^ expression. SQ ALT-803 alone resulted in significant increase in CD3/NKG2D expressing cells compared to PBS control

Two weeks after the final ALT-803 and/or BCG treatment, bladders were resected and stained for immune cell infiltrates (Fig. 3). At this timepoint, there was no difference in the number of tumor infiltrating CD4^+^ T cells, CD8^+^ T cells or CD161/KLRB1^+^ NK cells between the treatment groups and PBS control. Furthermore, no CD161/KLRB1^+^ NK cells were detected. It is conceivable that assessing tumors 2 weeks after the last treatment may not capture the infiltration of these immune cells. Next, whole blood from mice treated on this protocol collected 3 days after week 1 treatment was subjected to flow cytometric analysis for CD3^+^ (pan-T cell), CD3^+^/CD8^+^ (CD8^+^ T cell), NKG2D^+^ (NK) and CD3^+^/NKG2D^+^ (NKT) cells. Subcutaneous ALT-803 alone resulted in a robust induction of peripheral CD8^+^ T, NK and NKT cells compared to other groups. Interestingly, this induction of CD8^+^ T, NK and NKT cells by SQ ALT-803 alone was blunted with the addition of intravesical BCG (Fig. 4a). In the mice’s splenic tissue collected at this same time, only SQ ALT-803 alone resulted in a robust induction of NKT cells compared to PBS control (Fig. 4b). Splenic tissue collected at week 20 demonstrated that SQ ALT-803 with intravesical BCG resulted in induction of CD8^+^ T, NK and NKT cells (Additional file 1: Figure S1). Previously we noted minimal systemic effects with intravesical ALT-803 with or without intravesical BCG [6].

### Assessment of therapeutic induction of serum and urinary cytokines

**Fig. 5:**
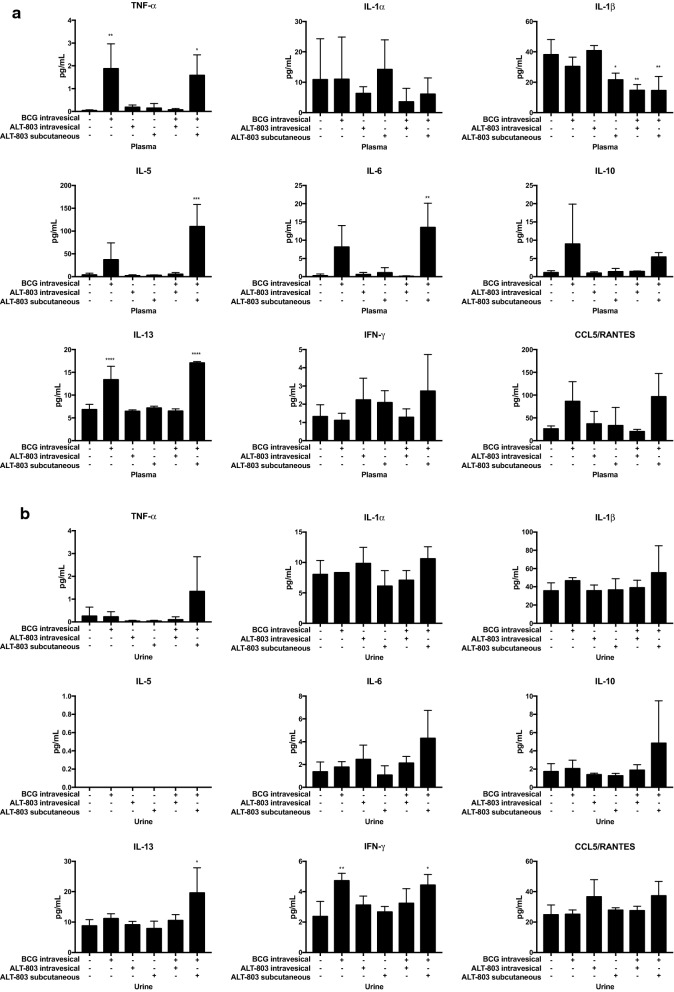
Changes in serum and urinary cytokine profiles following treatment of tumor bearing mice. **a** Serum cytokines—Serum samples collected 3 days after week 1 treatment from female mice bearing tumors were compared among the six treatment groups. Serum levels of a panel of cytokines were measured by the 9-plex customized mouse cytokine Luminex assay (R&D Systems). IVe BCG treatment alone was noted to induce TNFα and IL13 compared to PBS control. Combination SQ ALT-803 and IVe BCG was noted to induce TNFα, IL5, IL6 and IL13 compared to PBS control. Interestingly, ALT-803 SQ alone, IVe BCG + IVe ALT-803 and IVe BCG + SQ ALT-803 resulted in a reduction in interleukin 1β. **b** Urine cytokines—Voided urine samples collected 3 days after week 1 treatment from female mice bearing tumors were compared among the six treatment groups. Urine levels of a panel of cytokines were measured by the 9-plex customized mouse cytokine Luminex assay. IVe BCG alone resulted in an induction of IFNγ compared to PBS control. However, IVe BCG + SQ ALT-803 resulted in an induction of IL13 and IFNγ

Sera from mice treated on this protocol were collected 3 days after week 13 and at week 20 and immediately frozen at − 80 °C. Sera samples from week 13 were then thawed and subjected to analysis with the 9-plex customized mouse cytokine Luminex assay. Intravesical BCG treatment alone was noted to induce TNFα (p < 0.01) and IL13 (p < 0.001) compared to PBS control. There was a trend of elevated IL5, IL6, IL10, IL13 and RANTES in Intravesical BCG alone group compared to PBS control. Combination of SQ ALT-803 with intravesical BCG (not intravesical ALT-803) was noted to induce TNFα (p < 0.05), IL5 (p < 0.005), IL6 (p < 0.01) and IL13 (p < 0.005) compared to PBS control. There was a trend of elevated IL10 and RANTES in SQ ALT-803 with intravesical BCG compared to PBS control. The induction of IL5 and IL6 with SQ ALT-803 with intravesical BCG was superior compared to intravesical BCG alone. Interestingly, SQ ALT-803 alone (p < 0.05), intravesical BCG with intravesical ALT-803 (p < 0.01) and intravesical BCG plus SQ ALT-803 (p < 0.01) resulted in a reduction in IL1β (Fig. 5a). Sera cytokine profile at week 20 differed in that previously induced cytokines were no longer highly expressed (Additional file 2: Table S1).

Urine samples from mice treated on this protocol 3 days after week 13 and at 20 weeks were collected, immediately frozen at − 80 °C and stored. When ready for analysis, urine samples collected 3 days after week 13 were thawed and subjected to the 9-plex customized mouse cytokine Luminex assay. Intravesical BCG alone resulted in an induction of IFNγ compared to PBS control (p < 0.01). There was a trend of RANTES induction in the intravesical ALT-803 treatment group. Intravesical BCG with intravesical ALT-803 treatment did not result in an induction of any urine cytokines in this model. However, intravesical BCG with SQ ALT-803 treatment resulted in an induction of IL13 (p < 0.05) and IFNγ (p < 0.05) (Fig. 5b). There was a trend of elevated TNFα, IL6, IL10 and RANTES in the intravesical BCG treatment group compared to PBS control. Urine cytokine profile at week 20 differed in that previously induced cytokines were no longer highly expressed, while ALT-803 SQ noted high expression of IL13 (Additional file 2: Table S1).

## Discussion

IL2 and its closely related IL15 are cytokines capable of stimulating immune cells via interactions with shared signaling receptor components, IL2Rβ and IL2RγC. Specifically, IL15, a potent stimulant of CD8^+^ T cells and natural killer (NK) cells [18, 19], is a promising cancer immunotherapeutic agent being explored by several companies. ALT-803, the lead IL15 analogue in the field, is a complex of an IL15 superagonist N72D mutant and a dimeric IL15 receptor α Su/IgG1 Fc fusion protein that was found to exhibit enhanced biologic activity and potent anti-tumor efficacy in vivo with a substantially longer serum half-life than recombinant IL15 [20–22]. These preliminary findings indicate that this IL15 superagonist complex could serve as an ideal immunostimulatory cancer therapeutic.

In fact, ALT-803 has entered the clinic in a series of early phase clinical trials. Wrangle et al. reported ALT-803 in combination with nivolumab to be safe to administer in an outpatient setting in patients with advance non-small cell lung cancer. The recommended phase 2 dose of ALT-803 was noted to be 20 μg/kg given once per week subcutaneously (SQ) in combination with 240 mg intravenous nivolumab every 2 weeks [9]. Similarly, Romee et al. reported ALT-803 alone was well tolerated in patients who relapsed > 60 days after allogeneic hematopoietic cell transplantation. The recommended phase 2 dose of ALT-803 in this setting was 10 μg/kg intravenously or subcutaneously weekly for 4 consecutive weeks [11]. In our, phase Ib study of ALT-803 and BCG administered intravesically in BCG-naïve patients with NMIBC, we demonstrated that the combination therapy was well tolerated and the phase 2 recommended dose of ALT-803 was 400 μg/instillation [8].

Previously, we published the efficacy of ALT-803 intravesical therapy in a carcinogen induced rat model [6]. In order to further evaluate the possible advantage of SQ injection of ALT-803, as noted in two phase I clinical trials, we set out to compare the biological activity of SQ ALT-803 and intravesical ALT-803 with or without intravesical BCG. We assessed the anti-tumor efficacy and immune stimulation of each regimen. All treatments demonstrated similar anti-tumor efficacy and SQ ALT-803 alone and intravesical BCG plus SQ ALT-803 showed non-inferiority to standard of care, intravesical BCG alone. All treatment regimens were well tolerated. Notably, SQ ALT-803, when administered alone, activated CD8^+^ T, NK and NKT cells in the PBMC while activating NKT cells in the spleen. ALT-803 has been shown to stimulate the activation, proliferation, and expansion of CD8^+^ T cells and NK cells in rodent models [22], in cynomolgus monkeys [23] and in human phase 1 studies [9–11]. Unlike our previous study in which a rat carcinogen induced model was used [6], we were unable to demonstrate a statistically significant difference in the number of CD8^+^ T and/or NK cells infiltrating the tumor and bladder, though there was a trend of increased CD8^+^ T cells in the intravesical BCG with SQ ALT-803 treatment group. The difference between our results in rats and mice may be multifactorial. First, although the two models are rodents, they have been known to exhibit biological and molecular differences [24]. Second, we assessed tumor infiltrating cells 2 weeks after completion of therapy in the current mouse model and 1 week after the last treatment in the rat model which may not be the appropriate time point. Third, a larger number of animals in each treatment group may have been required to show a significant statistical difference (i.e., the study was powered for changes in tumor size not infiltration of immune cells). Interestingly, serum cytokines were significantly elevated with intravesical BCG treatment alone (i.e., TNFα and IL13) as well in SQ ALT-803 with intravesical BCG treatment group (TNFα, IL5, IL6 and IL13). Urine cytokines were significantly elevated with intravesical BCG treatment alone (IFNγ) as well as with SQ ALT-803 with intravesical BCG group (IL13 and IFNγ). Therefore, the data suggest that systemic administration of ALT-803 resulted in systemic changes, notably increased numbers of CD8^+^ T, NK and NKT cells within the peripheral blood and spleen as well as induction of key cytokines, *e.g.,* IL5, IL6 in the sera and IL13 and IFNγ in the urine compared to only intravesical BCG administration. Thus, it is possible that systemic ALT-803 therapy may result in a broader activation of CD8^+^ T and NK cells as well as NKT cells compared to intravesical ALT-803, which could translate into a greater potential to effectively eradicate extravesical tumor (i.e., metastatic disease to lymph node or distant tissues). This concept of eliciting a systemic immune response is being tested in the current clinical trial entitled “Different strains of BCG with or without vaccine in high grade NMIBC” (NCT 03091660) in which intravesical BCG is administered with or without SQ BCG vaccine. It is speculated that SQ BCG vaccine will elicit a broad systemic immune response, which would supplement the local immune response. As we and others have noted, BCG elicits a broad, non-specific immune response [25]. We offer the idea that SQ ALT-803 is ideal in this setting to elicit a long-term CD8^+^ T cell, NK cell and NKT cell responses both systemically and locally.

## Conclusions

Our results provide strong evidence that ALT-803 administered SQ is non-inferior to other treatment regimens, including intravesical BCG, which is standard of care in this setting. Subcutaneous administration of ALT-803 alone was associated with activation of CD8^+^ T cells, NK cells and NKT cells, while the combination of SQ ALT-803 and intravesical BCG resulted in induction of serum TNFα, IL5, IL6 and IL13 and urinary IL13 and IFNγ. Thus, it is postulated that SQ ALT-803 alone or in combination with intravesical BCG may be associated with stronger or pronounced CD8^+^ T cell, NK cell and NKT cell activation resulting in prolonged anti-tumor effects.

## Additional files

**Additional file 1: Figure S1.** Splenocytes collected 20 weeks after week 1 treatment were isolated and analyzed by flow cytometry for expression of CD3^+^, CD3^+^/CD8^+^, NKG2D^+^ and CD3^+^/NKG2D^+^ expression. BCG plus SQ ALT-803 resulted in significant increase in CD3/CD8, NKG2D and CD3/NKG2D expressing cells compared to PBS control.

**Additional file 2: Table S1.** Changes in plasma cytokine/chemokine profiles at week 20. **Table S2.** Changes in urinary cytokine/chemokine profiles at week 20.
