# Supplementary figures and images for: Effectiveness of two different dose administration regimens of an IL-15 superagonist complex (ALT-803) in an orthotopic bladder cancer mouse model
Source: J Transl Med. 2019 Jan 17;17:29. doi: 10.1186/s12967-019-1778-6 (PMC6337786; doi:10.1186/s12967-019-1778-6)

## Slide 1
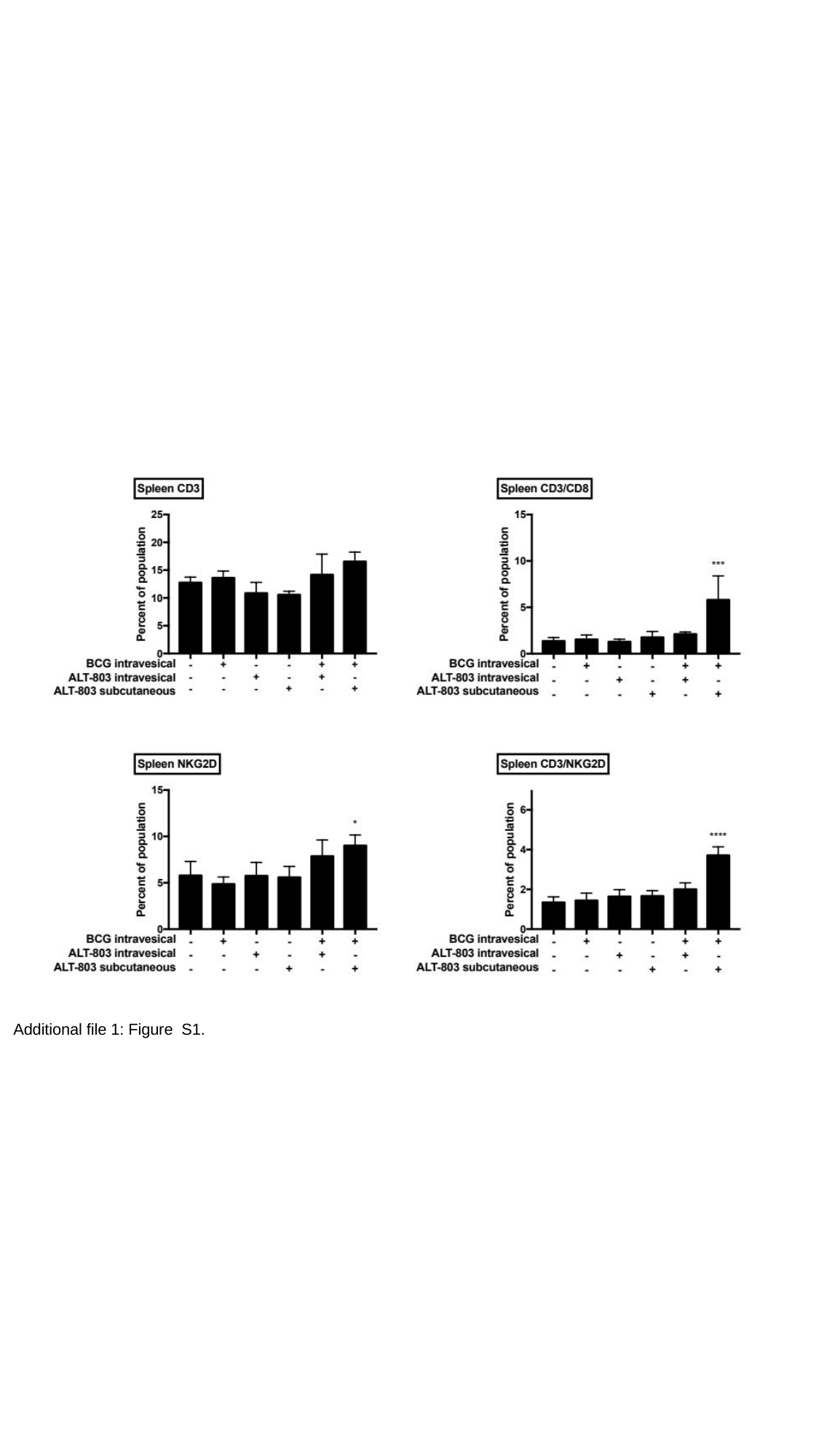

Additional file 1: Figure S1.

Supplement: Supplementary file 1 — Additional file 1: Figure S1. Splenocytes collected 20 weeks after week 1 treatment were isolated and analyzed by flow cytometry for expression of CD3+, CD3+/CD8+, NKG2D+ and CD3+/NKG2D+ expression. BCG plus SQ ALT-803 resulted in significant increase in CD3/CD8, NKG2D and CD3/NKG2D expressing cells compared to PBS control. [file 12967_2019_1778_MOESM1_ESM.pptx]
