# Supplementary material for: Effectiveness of two different dose administration regimens of an IL-15 superagonist complex (ALT-803) in an orthotopic bladder cancer mouse model
Source: J Transl Med. 2019 Jan 17;17:29. doi: 10.1186/s12967-019-1778-6 (PMC6337786; doi:10.1186/s12967-019-1778-6)
Supplement: Supplementary file 2 — Additional file 2: Table S1. Changes in plasma cytokine/chemokine profiles at week 20. Table S2. Changes in urinary cytokine/chemokine profiles at week 20. [file 12967_2019_1778_MOESM2_ESM.docx]

Table S1. Changes in plasma cytokine/chemokine profiles at week 20.

|  | PBS | | BCG | | ALT-803 IVe | | ALT-803 SQ | | BCG + ALT-803 IVe | | BCG + ALT-803 SQ | |  |
| --- | --- | --- | --- | --- | --- | --- | --- | --- | --- | --- | --- | --- | --- |
| **Th1** | |  | |  | |  | |  | |  | |  | |
| IL-2 | | 9.93 ± 3.31 | | 8.93 ± 3.01 | | 9.01 ± 1.52 | | ***4.59 ± 2.74***** | | ***5.02 ± 3.45**** | | 7.66 ± 1.16 | |
| IFN-γ | | 11.99 ± 6.13 | | 10.94 ± 5.82 | | 7.01 ± 1.67 | | ***5.75 ± 0.76**** | | ***5.96 ± 1.09**** | | 6.46 ± 1.74 | |
| TNF-α | | 26.56 ± 4.70 | | 31.17 ± 12.99 | | ***22.49 ± 1.90******* | | ***18.81 ± 1.63******* | | ***19.51 ± 1.08******* | | ***19.34 ± 1.20******* | |
|  | |  | |  | |  | |  | |  | |  | |
| **Th2** | |  | |  | |  | |  | |  | |  | |
| IL-4 | | 26.89 ± 44.23 | | 28.25 ± 47.10 | | 6.45 ± 15.81 | | 0.00 ± 0.00 | | 0.00 ± 0.00 | | 0.00 ± 0.00 | |
| IL-5 | | 132.17 ± 184.70 | | 120.86 ± 158.64 | | 24.19 ± 18.38 | | 10.62 ± 13.69 | | 13.44 ± 21.69 | | 11.16 ± 11.86 | |
| IL-6 | | 29.13 ± 30.47 | | 31.22 ± 45.04 | | 40.19 ± 27.19 | | 1.18 ± 2.78 | | 8.39 ± 10.12 | | 3.38 ± 4.11 | |
| IL-10 | | 0.00 ± 0.00 | | 0.00 ± 0.00 | | 0.00 ± 0.00 | | 0.00 ± 0.00 | | 0.00 ± 0.00 | | 0.00 ± 0.00 | |
| IL-13 | | 25.47 ± 22.04 | | 11.46 ± 17.82 | | 6.07 ± 14.87 | | 5.58 ± 8.64 | | ***0.00 ± 0.00**** | | ***0.00 ± 0.00**** | |
|  | |  | |  | |  | |  | |  | |  | |
| **Th17** | |  | |  | |  | |  | |  | |  | |
| IL-17 | | 7.16 ± 10.40 | | 5.66 ± 7.31 | | 1.43 ± 1.11 | | 0.60 ± 1.35 | | 1.10 ± 1.51 | | 0.89 ± 0.91 | |
|  | |  | |  | |  | |  | |  | |  | |
| **Inflammatory** | |  | |  | |  | |  | |  | |  | |
| IL-1α | | 0.00 ± 0.00 | | 2.29 ± 3.70 | | 4.28 ± 6.91 | | 0.00 ± 0.00 | | 0.00 ± 0.00 | | 0.00 ± 0.00 | |
| IL-1β | | 271.16 ± 394.48 | | 252.35 ± 312.98 | | 4.27 ± 10.47 | | 8.55 ± 13.24 | | 0.00 ± 0.00 | | 4.27 ± 10.47 | |
| IL-6 | | 29.13 ± 30.47 | | 31.22 ± 45.04 | | 40.19 ± 27.19 | | 1.18 ± 2.78 | | 8.39 ± 10.12 | | 3.38 ± 4.11 | |
| TNF-α | | 26.56 ± 4.70 | | 31.17 ± 12.99 | | ***22.49 ± 1.90******* | | ***18.81 ± 1.63******* | | ***19.51 ± 1.08******* | | ***19.34 ± 1.20******* | |
| IFN-γ | | 11.99 ± 6.13 | | 10.94 ± 5.82 | | 7.01 ± 1.67 | | ***5.75 ± 0.76**** | | ***5.96 ± 1.09**** | | 6.46 ± 1.74 | |
| GM-CSF | | 8.34 ± 5.22 | | 9.40 ± 10.59 | | 2.79 ± 4.33 | | 0.00 ± 0.00 | | 0.00 ± 0.00 | | 4.87 ± 7.64 | |
| IP-10 (CXCL10) | | 20.07 ± 8.18 | | 13.76 ± 2.09 | | ***12.00 ± 1.15**** | | ***10.99 ± 4.16***** | | ***12.67 ± 1.65**** | | 15.16 ± 5.75 | |
| MCP1 (CCL2) | | 56.23 ± 40.08 | | 34.72 ± 43.15 | | ***6.94 ± 0.00***** | | ***2.31 ± 3.58***** | | ***3.47 ± 3.80***** | | ***1.16 ± 2.83***** | |
| MIP 1α (CCL3) | | 31.12 ± 20.81 | | 56.47 ± 48.01 | | 32.26 ± 27.28 | | 13.65 ± 17.99 | | 5.69 ± 6.23 | | 35.69 ± 42.13 | |
| KC (CXCL1) | | 60.35 ± 69.03 | | 0.00 ± 0.00 | | 23.14 ± 56.68 | | 17.35 ± 42.50 | | 0.00 ± 0.00 | | 0.00 ± 0.00 | |
| MIG (CXCL9) | | 13.36 ± 20.85 | | 0.00 ± 0.00 | | 4.48 ± 10.98 | | 0.00 ± 0.00 | | 0.00 ± 0.00 | | 0.00 ± 0.00 | |
|  | |  | |  | |  | |  | |  | |  | |
| **Anti-Inflammatory** | |  | |  | |  | |  | |  | |  | |
| IL-4 | | 26.89 ± 44.23 | | 28.25 ± 47.10 | | 6.45 ± 15.81 | | 0.00 ± 0.00 | | 0.00 ± 0.00 | | 0.00 ± 0.00 | |
| IL-10 | | 0.00 ± 0.00 | | 0.00 ± 0.00 | | 0.00 ± 0.00 | | 0.00 ± 0.00 | | 0.00 ± 0.00 | | 0.00 ± 0.00 | |
| IL-13 | | 25.47 ± 22.04 | | 11.46 ± 17.82 | | 6.07 ± 14.87 | | 5.58 ± 8.64 | | ***0.00 ± 0.00**** | | ***0.00 ± 0.00**** | |
| IL-20 (p40/p70) | | 48.74 ± 51.41 | | 47.52 ± 22.33 | | 10.37 ± 6.59 | | 15.79 ± 24.49 | | 18.78 ± 22.98 | | 25.67 ± 22.64 | |
|  | |  | |  | |  | |  | |  | |  | |
| **Angiogenesis** | |  | |  | |  | |  | |  | |  | |
| FGF basic | | 183.67 ± 98.00 | | 145.52 ± 82.78 | | 344.77 ± 372.42 | | 81.96 ± 23.69 | | 246.51 ± 309.86 | | 57.10 ± 35.44 | |
| VEGF | | 3.42 ± 1.76 | | 1.83 ± 1.59 | | 1.47 ± 2.15 | | ***0.74 ± 1.18**** | | ***0.22 ± 0.49***** | | ***0.43 ± 0.60***** | |

Data indicates mean ± SD. * < 0.05, ** < 0.01 and **** < 0.0001 *vs*. Control.

Table S2. Changes in urinary cytokine/chemokine profiles at week 20.

|  | PBS | BCG | ALT-803 IVe | ALT-803 SQ | BCG + ALT-803 IVe | BCG + ALT-803 SQ |
| --- | --- | --- | --- | --- | --- | --- |
| **Th1** |  |  |  |  |  |  |
| IL-2 | 14.71 ± 4.34 | 13.52 ± 2.37 | 13.22 ± 3.49 | 12.77 ± 2.41 | ***10.12 ± 1.01**** | 10.45 ± 1.03 |
| IFN-γ | 7.20 ± 0.69 | 8.61 ± 3.51 | 6.63 ± 0.98 | 7.06 ± 1.02 | 6.40 ± 1.18 | 6.44 ± 0.00 |
| TNF-α | 23.64 ± 2.46 | 43.88 ± 50.96 | 22.95 ± 2.79 | 23.32 ± 0.95 | 22.65 ± 2.00 | 22.50 ± 1.50 |
|  |  |  |  |  |  |  |
| **Th2** |  |  |  |  |  |  |
| IL-4 | 40.50 ± 8.61 | 57.81 ± 56.09 | 23.00 ± 22.84 | 45.61 ± 11.22 | 17.73 ± 19.75 | 13.89 ± 14.52 |
| IL-5 | 0.00 ± 0.00 | 0.64 ± 1.57 | 2.08 ± 3.56 | 4.65 ± 4.17 | 0.00 ± 0.00 | 2.73 ± 5.06 |
| IL-6 | 20.90 ± 15.12 | 15.62 ± 9.13 | 15.29 ± 7.57 | 15.49 ± 9.81 | ***4.99 ± 2.90**** | 7.70 ± 2.27 |
| IL-10 | 36.52 ± 10.78 | 44.84 ± 45.77 | 19.47 ± 24.30 | 26.50 ± 7.87 | 8.22 ± 11.00 | 3.88 ± 4.93 |
| IL-13 | 20.44 ± 16.42 | 41.41 ± 24.58 | 9.50 ± 10.70 | ***58.41 ± 16.48***** | 24.07 ± 18.72 | 6.50 ± 14.68 |
|  |  |  |  |  |  |  |
| **Th17** |  |  |  |  |  |  |
| IL-17 | 1.20 ± 0.72 | 3.22 ± 5.19 | 0.34 ± 0.48 | 1.35 ± 1.01 | 2.82 ± 5.34 | 1.10 ± 2.01 |
|  |  |  |  |  |  |  |
| **Inflammatory** |  |  |  |  |  |  |
| IL-1α | 95.00 ± 147.71 | 85.59 ± 36.77 | 110.70 ± 86.22 | 66.24 ± 75.76 | 14.26 ± 13.04 | 1.56 ± 1.47 |
| IL-1β | 71.78 ± 16.52 | 73.74 ± 67.31 | 38.50 ± 36.66 | 43.72 ± 15.88 | ***14.79 ± 22.91**** | 17.10 ± 13.24 |
| IL-6 | 20.90 ± 15.12 | 15.62 ± 9.13 | 15.29 ± 7.57 | 15.49 ± 9.81 | ***4.99 ± 2.90**** | 7.70 ± 2.27 |
| TNF-α | 23.64 ± 2.46 | 43.88 ± 50.96 | 22.95 ± 2.79 | 23.32 ± 0.95 | 22.65 ± 2.00 | 22.50 ± 1.50 |
| IFN-γ | 7.20 ± 0.69 | 8.61 ± 3.51 | 6.63 ± 0.98 | 7.06 ± 1.02 | 6.40 ± 1.18 | 6.44 ± 0.00 |
| GM-CSF | 23.72 ± 12.65 | 23.22 ± 19.63 | 10.79 ± 9.61 | 8.04 ± 10.00 | ***2.79 ± 4.33**** | ***4.19 ± 4.59**** |
| IP-10 (CXCL10) | 52.18 ± 16.46 | 41.31 ± 18.07 | 33.41 ± 30.36 | 32.40 ± 7.22 | ***19.01 ± 12.25**** | 27.26 ± 16.56 |
| MCP1 (CCL2) | 22.25 ± 9.58 | 26.49 ± 25.43 | 16.67 ± 4.77 | 10.83 ± 6.03 | 7.73 ± 6.01 | 10.83 ± 6.03 |
| MIP 1α (CCL3) | 156.38 ± 123.74 | 143.68 ± 49.56 | 150.37 ± 86.95 | 119.91 ± 73.95 | 48.12 ± 12.62 | ***40.41 ± 11.67**** |
| KC (CXCL1) | 480.89 ± 79.94 | 552.31 ± 73.86 | 376.54 ± 174.12 | 531.23 ± 38.10 | 351.95 ± 140.88 | 359.33 ± 67.05 |
| MIG (CXCL9) | 36.41 ± 20.30 | 84.76 ± 25.35 | 28.72 ± 24.34 | 79.18 ± 11.93 | 38.20 ± 36.29 | 94.80 ± 116.22 |
|  |  |  |  |  |  |  |
| **Anti-Inflammatory** |  |  |  |  |  |  |
| IL-4 | 40.50 ± 8.61 | 57.81 ± 56.09 | 23.00 ± 22.84 | 45.61 ± 11.22 | 17.73 ± 19.75 | 13.89 ± 14.52 |
| IL-10 | 36.52 ± 10.78 | 44.84 ± 45.77 | 19.47 ± 24.30 | 26.50 ± 7.87 | 8.22 ± 11.00 | 3.88 ± 4.93 |
| IL-13 | 20.44 ± 16.42 | 41.41 ± 24.58 | 9.50 ± 10.70 | ***58.41 ± 16.48***** | 24.07 ± 18.72 | 6.50 ± 14.68 |
| IL-20 (p40/p70) | 0.00 ± 0.00 | 2.16 ± 5.28 | 0.00 ± 0.00 | 0.00 ± 0.00 | 0.00 ± 0.00 | 0.00 ± 0.00 |
|  |  |  |  |  |  |  |
| **Angiogenesis** |  |  |  |  |  |  |
| FGF basic | 6.13 ± 13.71 | 2.12 ± 5.20 | 0.00 ± 0.00 | 2.12 ± 5.20 | 2.12 ± 5.20 | 14.57 ± 24.04 |
| VEGF | 450.02 ± 565.90 | 249.37 ± 44.63 | 296.99 ± 296.16 | 198.13 ± 59.86 | 92.65 ± 26.69 | 126.17 ± 25.60 |

Data indicates mean ± SD. * < 0.05 and ** < 0.01 *vs*. Control.
